# Assessment of lymph node status in gallbladder cancer: location, number, or ratio of positive nodes

**DOI:** 10.1186/1477-7819-10-87

**Published:** 2012-05-17

**Authors:** Yoshio Shirai, Jun Sakata, Toshifumi Wakai, Taku Ohashi, Yoichi Ajioka, Katsuyoshi Hatakeyama

**Affiliations:** 1 Division of Digestive and General Surgery, Niigata University Graduate School of Medical and Dental Sciences, 1-757 Asahimachi-dori, Chuo-ku, Niigata City 951-8510, Japan; 2 Division of Molecular and Diagnostic Pathology, Niigata University Graduate School of Medical and Dental Sciences, 1-757 Asahimachi-dori, Chuo-ku, Niigata City 951-8510, Japan

**Keywords:** Gallbladder neoplasms, Lymphatic metastasis, Lymph node ratio, Lymph node excision, Prognosis

## Abstract

**Background:**

Assessment of lymph node status is a critical issue in the surgical management of gallbladder cancer. The aim of this study was to compare the anatomical location of positive nodes, number of positive nodes, and lymph node ratio (LNR) as prognostic predictors in gallbladder cancer.

**Methods:**

We conducted a retrospective analysis of 135 patients with gallbladder cancer who underwent a radical resection with regional lymphadenectomy. A total of 2,245 regional lymph nodes were retrieved (median, 14 per patient). The location of positive nodes was classified according to the AJCC staging manual (7th edition). ‘Optimal’ cutoff values were determined for the number of positive nodes and LNR based on maximal *χ*^2^ scores calculated with the Cox proportional hazards regression model.

**Results:**

Lymph node metastasis was found histologically in 59 (44%) patients. The ‘optimal’ cutoff values for the number of positive nodes and LNR were determined to be three nodes and 10%, respectively. Univariate analysis identified location of positive nodes (pN0, pN1, pN2; *P* < 0.001), number of positive nodes (0, 1 to 3, ≥4; *P* < 0.001), and LNR (0%, 0 to 10%, >10%; *P* < 0.001) as significant prognostic factors. Multivariate analysis identified number of positive nodes as an independent prognostic factor ( *P* = 0.004); however, location of positive nodes and LNR failed to remain as an independent variable.

**Conclusions:**

The number of positive lymph nodes better predicts patient outcome after resection than either the location of positive lymph nodes or LNR in gallbladder cancer. Dividing the number of positive lymph nodes into three categories (0, 1 to 3, or ≥4) is valid for stratifying patients based on the prognosis after resection.

## Background

Lymph node status (nodal status) is an established prognostic factor in various gastrointestinal malignancies [1-7]. There are three conventional parameters describing nodal status: the anatomical location of positive lymph nodes [8,9], the number of positive lymph nodes [4,6,7], and the lymph node ratio (LNR; the ratio of the number of positive nodes to the number of nodes evaluated) [1-3].

In the cases of gallbladder cancer, both the American Joint Committee on Cancer (AJCC; 7th edition) [8] and the Japanese Society of Biliary Surgery [9] have subdivided the nodal status into three categories (N0, N1, or N2) and four categories (N0, N1, N2, or N3), respectively, according to the anatomical location of positive lymph nodes. In 2006, Endo *et al.*[10] first reported that the number of positive nodes better predicts the prognosis post-resection than the topographical location of positive nodes in patients with node-positive gallbladder cancer. In 2010, we also found that the number of positive nodes better predicted survival than the location of positive nodes (as defined by the Japanese Society of Biliary Surgery [9]) in patients undergoing an R0 resection [11]. However, in 2011, Negi *et al.*[12] were the first to report that LNR, but not the location or number of positive nodes, independently predicts survival after resection. Therefore, which of the three nodal status parameters best stratifies patients with gallbladder cancer according to prognosis remains controversial.

The current study compared the prognostic power of positive node location (as defined by the AJCC; 7th edition [8]), number of positive nodes, and LNR, by analyzing the long-term outcomes of 135 patients who underwent a radical resection for gallbladder cancer. Cutoff points for both the number of positive nodes and LNR were determined using *χ*^2^ scores calculated by the Cox proportional hazards regression model.

## Methods

### Patient population

From May 1982 to January 2009, 148 consecutive patients underwent a radical resection for gallbladder cancer in the study department, defined as a resection of both the primary tumor and regional lymph nodes. Thirteen patients with an invasive primary malignant tumor in other organs were excluded, leaving 135 patients for this retrospective study. They included 94 women and 41 men with ages ranging from 37 to 85 (median, 68) years.

### Radical resection procedures

A variety of radical resection procedures were performed in this series, with the choice of procedure based on the extent of tumor spread in a given patient (Table 1). An ‘extended’ radical cholecystectomy, which was instituted at our department in 1982, was the most common operation among our study cohort [11,13,14]; it involved a cholecystectomy, wedge resection of the gallbladder fossa with a rim of non-neoplastic liver tissue (about 2 cm in thickness or more), resection of a suprapancreatic segment of the extrahepatic bile duct, and *en bloc* regional lymph node dissection. Late-stage diseases often required more extensive resections such as major hepatectomy (defined as removal of two sections or more extended hepatectomy), pancreaticoduodenectomy (the Whipple procedure or pylorus-preserving procedure), or major hepatectomy combined with pancreaticoduodenectomy (Table 1) [11,14]. In contrast, some patients with early-stage disease, comorbid disease(s), or advanced age underwent a less aggressive resection, omitting the bile duct resection and/or a hepatectomy (Table 1). Although pathological T1 (pT1) tumors do not warrant radical resection [15], 21 patients with these tumors also underwent a radical resection because pT2 or more advanced disease was not ruled out before resection.

**Table 1:** Radical resection procedures for 135 patients with gallbladder cancer

**Procedure**	**Number of patients**
Extended cholecystectomy	
C + WR + BD + N*	53
C + WR + N	23
C† + N	11
C† + BD + N	6
More extensive resection	
C + ERH + BD + N	14
C + Central hepatectomy‡ + BD + N	3
C + ELH + BD + N	1
C + Right trisectionectomy + BD + N	1
C + WR + PD + N	15
C + ERH + PD + N	6
C + ERH + PPPD + N	2

*Designated as ‘extended’ radical cholecystectomy at our department since 1982 [11,13,14].

†Cholecystectomy with full-thickness dissection: cholecystectomy combined with removal of the cystic plate.

‡Defined as removal of Couinaud segments IV, V, and VIII.

C, cholecystectomy; WR, wedge resection of the gallbladder fossa; BD, resection of the extrahepatic bile duct; N, regional lymphadenectomy; ERH, extended right hepatectomy (right hepatectomy extended to an inferior portion of Couinaud segment 4); ELH, extended left hepatectomy (left hepatectomy extended to an inferior portion of right anterior section); PD, Whipple pancreaticoduodenectomy; PPPD, pylorus-preserving pancreaticoduodenectomy.

The cohort also included 18 patients who underwent a combined resection of contiguous tissues comprising the transverse colon (n = 11), duodenum (n = 4), portal vein (n = 3), stomach (n = 1), and inferior vena cava (n = 1). Among the total of 135 patients, 111 underwent an initial radical resection and 24 underwent a radical second resection after a prior simple cholecystectomy for presumed benign disease [16].

### Lymph node dissection procedures

The regional lymph nodes of the gallbladder included the cystic duct, pericholedochal, posterior superior (posterosuperior) pancreaticoduodenal, retroportal, right celiac, and hepatic artery node groups [11,14,17]. In most patients, these node groups were dissected *en bloc*. In the patients who underwent a pancreaticoduodenectomy, the right portion of the superior mesenteric node group was also dissected together with the above node groups. In some patients with early-stage disease, advanced age, or comorbid diseases, a less aggressive regional lymphadenectomy was performed at the discretion of the individual surgeons. In this series, 48 patients with suspected (or confirmed) regional nodal disease also underwent a dissection of the paraaortic lymph nodes (cephalad to the origin of the inferior mesenteric artery) [11,14,17].

### Pathological examination

All pathological findings were documented by using the AJCC cancer staging manual (7th edition) [8]. The primary tumor was classified as pT1 in 21 patients, pT2 in 59, pT3 in 36, and pT4 in 19. Adenocarcinoma was identified as the primary tumor in 114 patients, adenosquamous carcinoma in 18, squamous cell carcinoma in 2, and undifferentiated carcinoma in 1. Residual tumor status was judged as no residual tumor (R0) or microscopic/macroscopic residual tumor (R1/2).

Immediately after resection, the surgeon(s) retrieved lymph nodes from the node-bearing adipose tissues of the fresh surgical specimen, and grouped them according to location. A total of 2,829 lymph nodes (comprising 2,245 regional and 584 paraaortic nodes) were retrieved from the 135 patients. A representative section, 3-μm thick, was cut from each lymph node retrieved, and the nodes examined for metastases on routine histological examination using hematoxylin and eosin.

### Assessment of the nodal status

The number of positive lymph nodes as well as the total lymph node count (TLNC) was recorded for each patient. Paraaortic lymph nodes (if any) were not included in the TLNC, and any metastases detected in these lymph nodes were categorized as distant metastases and designated as pM1 [8]. Thus, in the current study, the number of positive lymph nodes did not include any positive paraaortic nodes detected.

Three parameters were used to assess the nodal status in individual patients: the location of positive lymph nodes, the number of positive lymph nodes, and LNR. The location of positive nodes was classified into three categories: pN0, pN1, pN2, according to the AJCC cancer staging manual (7th edition) [8]. LNR was calculated by dividing the number of positive nodes by the TLNC.

### Patient follow-up after resection

Three patients died post-resection during a hospital stay, giving an in-hospital mortality rate of 2%. Adjuvant treatment after resection was administered at the discretion of the individual surgeons. Thirty-six patients received oral administration of 5-fluorouracil or its derivatives. Eight patients received intravenous administration of 5-fluorouracil alone or in combination with other agents. Six patients received intravenous administration of gemcitabine. No patients received adjuvant radiotherapy.

Patients discharged home were followed regularly in outpatient clinics every one to six months for at least five years, with a median follow-up period of 146 (range, 1 to 332) months. At the time of disease status assessment, 53 patients had died of tumor recurrence and 19 patients had died of other causes with no evidence of tumor recurrence. One patient was alive with recurrent disease, and the remaining 62 patients were alive without the disease.

### Statistical analysis

Medical records and survival data were obtained for all patients. The survival time in each patient was defined as the interval between the date of the definitive resection and the date of the last follow-up or death. Only deaths from tumor recurrence were treated as failure cases in the analysis of disease-specific survival (DSS). The Kaplan-Meier method was used to estimate cumulative DSS rates, and the log rank test was used to evaluate differences between groups.

For the number of positive lymph nodes, LNR, and TLNC, the ‘optimal’ cutoff values were determined using *χ*^2^ scores, which were calculated using the Cox proportional hazards regression model. Eleven conventional variables (gallstone, type of radical resection, timing of radical resection, pT classification, pM classification, histological type, histological grade, lymphatic vessel invasion, venous invasion, perineural invasion, and residual tumor status) were found to be significant by univariate analysis (the log rank test; Table 2), and these were entered as covariates in the model. The maximal *χ*^2^ scores indicate the ‘optimal’ cutoff values [5,18,19].

**Table 2:** Factors influencing long-term survival after resection in 135 patients with gallbladder cancer

**Variable**	**Number of patients**	**Survival rate (%)**		**Univariate analysis**	**Multivariate analysis**	
		**5-year**	**10-year**	***P* value**	**Relative risk (95% CI)**	***P* value**
Age (years)				0.123		
≤70	86	66	63			
>70	49	52	49			
Sex				0.652		
Male	41	66	61			
Female	94	59	57			
Gallstone				0.028		0.003
Absent	73	52	50		2.801 (1.435-5.470)	
Present	62	72	68		1.000	
Type of radical resection				<0.001		
Extended cholecystectomy	93	77	76			
Major hepatectomy	19	9	9			
Pancreaticoduodenectomy	15	45	28			
Major hepatectomy with pancreaticoduodenectomy	8	25	25			
Timing of radical resection				0.007		
Initial radical resection	111	56	52			
Radical second resection	24	86	86			
Adjuvant chemotherapy				0.093		
Absent	85	57	52			
Present	50	68	68			
Size of the primary tumor (mm)				0.869		
≤60	68	61	59			
>60	67	61	57			
pT classification*				<0.001		0.005
pT1 plus pT2	80	87	85		1.000	
pT3 plus pT4	55	21	15		3.145 (1.412-6.948)	
Total lymph node count (TLNC)				0.102		
≤16	76	66	66			
>16	59	55	48			
Location of positive lymph nodes*				<0.001		
pN0	76	80	80			
pN1	24	57	46			
pN2	35	23	20			
Number of positive lymph nodes				<0.001		0.004
0	76	80	80		1.000	
1-3	39	51	41		1.640 (0.784-3.431)	
≥4	20	10	10		4.997 (1.905-13.003)	
Lymph node ratio (LNR; %)				<0.001		
0	76	80	80			
0-10	22	60	49			
>10	37	24	20			
pM classification*				<0.001		0.014
pM0	109	72	69		1.000	
pM1	26†	13	7		2.260 (1.178-4.337)	
Histological type*				<0.001		
Adenocarcinoma	114	66	64			
Others	21	32	26			
Histological grade*				0.001		<0.001
G1 plus G2	96	68	67		1.000	
G3 plus G4	39	42	34		3.472 (1.766-6.828)	
Lymphatic vessel invasion (L)*				<0.001		
L0	57	85	83			
L1	78	44	41			
Venous invasion (V)*				<0.001		0.007
V0	74	78	76		1.000	
V1	61	41	37		2.445 (1.279-4.673)	
Perineural invasion				<0.001		
Absent	88	77	74			
Present	47	29	26			
Residual tumor status*				<0.001		0.003
R0	121	67	64		1.000	
R1 plus R2	14	0	0		3.306 (1.519-7.195)	

*According to the AJCC cancer staging manual (7th edition) [8].

†Includes 13 with paraaortic nodal disease.

CI, confidence interval; pT classification, pathological primary tumor classification; pN classification, pathological regional lymph nodes classification; pM classification, pathological distant metastasis classification; G1, well differentiated; G2, moderately differentiated; G3, poorly differentiated; G4, undifferentiated; L0, no lymphatic vessel invasion; L1, lymphatic vessel invasion; V0, no venous invasion; V1, microscopic venous invasion; R0, no residual tumor; R1, microscopic residual tumor; R2, macroscopic residual tumor.

To determine factors influencing long-term survival after resection, 15 conventional variables together with TLNC, the location of positive nodes, the number of positive nodes, and LNR were tested in the 135 patients (Table 2). The Cox proportional hazards regression model using a step-forward fitting procedure was applied to identify independent factors associated with survival. In this model, a step-wise selection was used for variable selection with entry and removal limits of *P* < 0.05 and *P* > 0.10, respectively.

The IBM SPSS Statistics 19 software (IBM Japan, Tokyo, Japan) was used for all statistical evaluations. All tests were two-tailed, and *P* values < 0.05 were taken to indicate statistical significance.

## Results

A total of 2,245 regional lymph nodes were retrieved from the 135 patients, with TLNC per patient ranging from 3 to 55 (median, 14). Of the study patients, 59 (44%) had a total of 252 positive lymph nodes; the number of positive nodes ranged from 1 to 26 (median, 2) per patient, and LNR ranged from 2.6% to 93% (median; 13%).

### Cutoff values for the number of positive lymph nodes, lymph node ratio, and total lymph node count

Tables 3 and 4 depict the analysis of number of positive nodes and LNR by the Cox proportional hazards regression model. Based on the maximal *χ*^2^ score, the ‘optimal’ cutoff value was three nodes for the number of positive nodes (Table 3) and 10% for LNR (Table 4). Similarly, the estimated ‘optimal’ cutoff value for TLNC was 16 nodes (data not shown). Based on these results, both the number of positive nodes and LNR were placed into one of three categories in subsequent analyses (0, 1 to 3, or ≥4 and 0%, 0 to 10%, or >10%, respectively), while TLNC was divided into two categories (≤16 or > 16).

**Table 3:** Analysis of the number of positive lymph nodes using the Cox proportional hazards regression model

**Cutoff value for positive node number**	***χ***^**2**^**score**	***P* value**
0, ≥1	5.217	0.022
0, 1, ≥2	7.832	0.020
0, 1–2, ≥3	7.611	0.022
0, 1–3, ≥4	13.234	0.001
0, 1–4, ≥5	7.213	0.027
0, 1–5, ≥6	5.275	0.072
0, 1–6, ≥7	5.275	0.072
0, 1–7, ≥8	5.233	0.073

**Table 4:** Analysis of the lymph node ratio (LNR) calculated by the Cox proportional hazards regression model

**Cutoff value for LNR (%)**	***χ***^**2**^**score**	***P* value**
0, 0–5, >5	7.006	0.030
0, 0–10, >10	7.547	0.023
0, 0–15, >15	6.516	0.038
0, 0–20, >20	6.731	0.035
0, 0–25, >25	6.881	0.032
0, 0–30, >30	6.212	0.045
0, 0–35, >35	6.844	0.033
0, 0–40, >40	5.938	0.051

### Factors influencing disease-specific survival after resection

Univariate analyses identified gallstone, type of radical resection, timing of radical resection, pT classification, location of positive lymph nodes (pN0, pN1, pN2), number of positive lymph nodes (0, 1 to 3, ≥4), LNR (0%, 0 to 10%, >10%), pM classification, histological type, histological grade, lymphatic vessel invasion, venous invasion, perineural invasion, and residual tumor status as significant prognostic factors (Table 2). TLNC failed to significantly affect DSS.

The univariately significant variables were then entered into multivariate analyses, with gallstone, pT classification, number of positive lymph nodes, pM classification, histological grade, venous invasion, and residual tumor status remaining as independently significant variables (Table 2). Neither location of positive lymph nodes nor LNR were identified as an independent variable by multivariate analysis.

### Impact of total lymph node count on disease-specific survival after resection

DSS after resection did not differ significantly between the 76 patients with TLNC ≤ 16 (median DSS, not reached; 5-year DSS, 66%) and the 59 patients with TLNC > 16 (median DSS, 77 months; 5-year DSS, 55%) (*P* = 0.102; Table 2).

We then focused on a subgroup of 76 patients without nodal disease (pN0) for further survival analysis. Even in this subgroup of patients, no significant difference in DSS was noted between 51 with TLNC ≤ 16 (median DSS, not reached; 5-year DSS, 80%) and 25 with TLNC > 16 (median DSS, not reached; 5-year DSS, 82%) (*P* = 0.707).

### Impact of nodal status on disease-specific survival after resection

**Figure 1:**
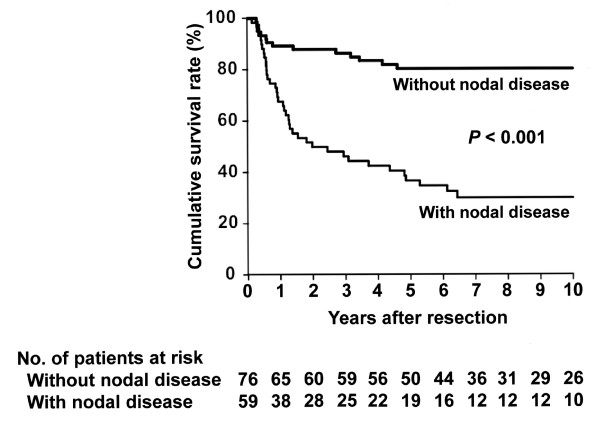
**Kaplan-Meier disease-specific survival estimates according to the presence or absence of regional nodal disease.** The median survival time was not reached with a 5-year survival rate of 80% in patients without nodal disease, whereas the median survival time was 24 months with a 5-year survival rate of 37% in patients with nodal disease ( *P* < 0.001).

**Figure 2:**
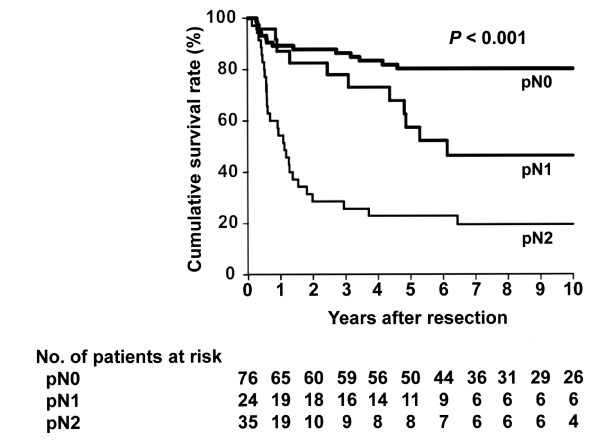
**Kaplan-Meier disease-specific survival estimates according to the location of positive regional lymph nodes.** The median survival time was not reached with a 5-year survival rate of 80% in patients without nodal disease (pN0). The median survival time was 74 months with a 5-year survival rate of 57% in patients with pN1 disease. The median survival time was 13 months with a 5-year survival rate of 23% in patients with pN2 disease. The survival post-resection differed significantly among the groups ( *P* < 0.001).

**Figure 3:**
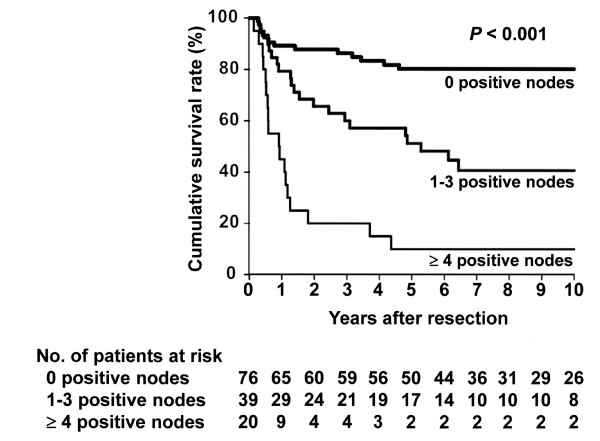
**Kaplan-Meier disease-specific survival estimates according to the number of positive regional lymph nodes.** The median survival time was not reached with a 5-year survival rate of 80% in patients without nodal disease. The median survival time was 63 months with a 5-year survival rate of 51% in patients with 1 to 3 positive nodes. The median survival time was 11 months with a 5-year survival rate of 10% in patients with ≥4 positive nodes. The survival post-resection differed significantly among the groups ( *P* < 0.001).

**Figure 4:**
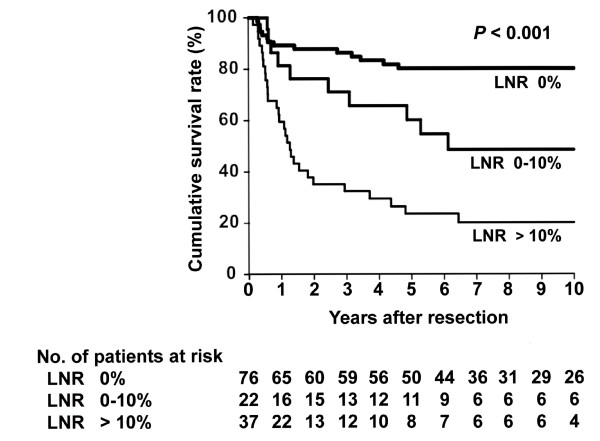
**Kaplan-Meier disease-specific survival estimates according to the lymph node ratio (LNR) of regional lymph nodes.** The median survival time was not reached with a 5-year survival rate of 80% in patients without nodal disease (LNR of 0%). The median survival time was 74 months with a 5-year survival rate of 60% in patients with a LNR of 0 to 10%. The median survival time was 15 months with a 5-year survival rate of 24% in patients with a LNR of > 10%. The survival post-resection differed significantly among the groups ( *P* < 0.001).

Nodal disease was significantly associated with lower DSS in this series (*P* < 0.001; Figure 1). When the total group of 135 patients was stratified based on location of positive lymph nodes, 76 had pN0 disease, 24 had pN1 disease, and 35 had pN2 disease, revealing that DSS after resection differed significantly among the groups (Figure 2). When divided into three groups according to the number of positive lymph nodes, 76 had 0 positive nodes, 39 had 1 to 3 positive nodes, and 20 had ≥4 positive nodes, revealing that DSS after resection also differed significantly among these groups (Figure 3). Finally, groups also differed significantly in DSS after resection when divided according to LNR; 76 had a ratio of 0%, 22 had a ratio of 0 to 10%, and 37 had a ratio of >10% (Figure 4).

As shown in Table 2, among the three parameters representing nodal status, only the number of positive lymph nodes (0, 1 to 3, ≥4) was found to be an independent prognostic factor.

## Discussion

Adequate assessment of the nodal status is a critical issue in the surgical management of patients with gallbladder cancer [10-12,18]. However, among the three conventional parameters of nodal status, which are location of positive lymph nodes, number of positive lymph nodes, and LNR, what best stratifies the patients with gallbladder cancer remains unresolved and prompted the current study. Here, we demonstrated that only the number of positive lymph nodes, and not location or LNR, independently affected outcomes after resection in our study cohort, suggesting that the number of positive lymph nodes is a potent parameter in assessing the nodal status of gallbladder cancer [10,11].

The AJCC cancer staging manual (6th edition) recommended ‘analysis of a minimum of three lymph nodes’ for accurate staging of gallbladder cancer [20]. However, recent population-based studies by Coburn *et al*. [21] and Mayo *et al*. [22] disclosed that among patients in the United States with resectable gallbladder cancer, only 5.3% to 6.9% had lymphadenectomy of ≥3 lymph nodes. In 2011, a report from the Memorial Sloan-Kettering Cancer Center of 122 patients who underwent a portal lymph node dissection cited a median TLNC of only 3 nodes [18]. The above observations suggest that compliance with the AJCC recommendation of retrieving a minimum of three lymph nodes remains poor in the United States. In general, retrieval of only a few lymph nodes may leave behind metastatic positive lymph nodes, which could result in recurrent disease as well as underestimation (that is,, downstaging) of the nodal status [1-5,12,18].

In contrast, Japanese hepatobiliary surgeons, including us, maintain an aggressive attitude toward regional lymphadenectomy for gallbladder cancer [10,11,13,14,23]. As a result of retrieving a large number of lymph nodes, 5-year survival statistics in patients with nodal disease have improved according to the Japanese literature [10,11,13,14,23]. In the current series, retrieval of a large number of regional nodes (median of 14 nodes) yielded 19 individuals with nodal disease alive after 5 years (Figure 1). Coburn *et al*. [21] and Mayo *et al*. [22] independently associated lymphadenectomy accompanied by evaluation of ≥3 lymph nodes with improved survival among T2 and T3 patients. Also, Ito *et al*. [18] and Negi *et al*. [12] independently suggested that retrieval and evaluation of at least six lymph nodes improves risk-stratification after resection in node-negative patients. Taken together, the above observations indicate that retrieval of a larger number of lymph nodes than previously practiced (for example, ≥6 nodes [12,18]) is warranted not only for accurately staging the nodal status, but also for improving survival due to better clearance of nodal disease. We believe that adequate lymphadenectomy is indispensable for improving the prognosis post-resection in patients with gallbladder cancer [14].

In various gastrointestinal malignancies, evaluating a limited number of lymph nodes may result in an underestimated number of positive nodes, leading to ‘stage migration’ (that is, downstaging) [1-5,12,18]. To solve this issue, many investigators have advocated LNR, which is defined as the number of positive nodes divided by TLNC [1-3,12]. LNR is of particular value in patients who cannot adequately be staged because of the limited number of lymph nodes evaluated [1-3]. Thus, in the case of insufficient lymph node evaluation, LNR will more accurately reflect the nodal status than the number of positive nodes, as shown for various malignancies [1-3]. Even in gallbladder cancer, Negi and colleagues [12] first found that LNR, and not the number of positive nodes, was an independent prognostic factor in their study cohort comprising 57 patients with a relatively small TLNC (median of 5 in node-negative patients; 6 in node-positive patients). Conversely, provided that lymph node evaluation is sufficient, such stage migration will be minimized, and thus the number of positive nodes will more directly reflect the nodal status than LNR. This was independently confirmed by Murakami *et al*. [4], Lee *et al*. [7], Sakata *et al*. [19], and Sierzega *et al*. [24] for pancreaticobiliary malignancies. Thus, the sufficient evaluation of regional lymph nodes in our series (median; 14 nodes per patient) may partly explain why the number of positive lymph nodes better stratified the patients than LNR.

Regarding gallbladder cancer, Endo *et al*. [10] divided the number of positive lymph nodes into two categories (1 or ≥2), probably due to no 5-year survivors with multiple positive nodes in their study cohort. In a previous study [11], we arbitrarily divided the number of positive nodes into four categories (0, 1, 2 to 3, or ≥4). In these two studies, however, cutoff point analysis was not performed for the number of positive nodes. Although several statistical methods are used for cutoff point analysis [1-3,12,19,24], the current study used *χ*^2^ scores calculated by the Cox proportional hazards regression model (Table 3) and thus determined the cutoff value to be three positive nodes. We believe that dividing the number of positive nodes into three categories (0, 1 to 3, or ≥4) is valid for stratifying patients with gallbladder cancer according to prognosis after resection (Figure 3), although this observation may be applicable only in those cases where sufficient lymph node evaluation (that is, adequate lymphadenectomy) is performed, as in the current series.

Both the AJCC cancer staging manual (7th edition) [8] and the Japanese Society of Biliary Surgery [9] categorize nodal status based on the anatomical location of positive nodes for gallbladder cancer. In 2006, Endo *et al*. [10] suggested that the number of positive nodes is more useful in assessing nodal status than the location of positive nodes. In 2010, we additionally showed that the number, but not location (as defined by the Japanese Society of Biliary Surgery [9]), of positive nodes independently determined prognosis after resection. In the current study, the number of positive nodes better predicted prognosis post-resection than the location of positive nodes (as defined by the AJCC cancer staging manual; 7th edition [8]). In addition, the location of lymph nodes is practically difficult to determine in *en bloc* resected specimens. Therefore, the number rather than the location of positive nodes appears to be a more useful parameter of nodal status in gallbladder cancer.

The current study has several limitations: the retrospective nature of the analysis, the relatively small number of patients spanning a long period of time, some variability in the degree of nodal dissection, and the short follow-up time for some patients. We believe, however, that these limitations did not greatly affect the results of the study as the differences between groups were too marked to have resulted from bias. In addition, the role of the number of positive nodes in assessing the nodal status for gallbladder cancer is now more clearly defined than previously based on the current study. Our results thus provide useful information for accurately staging nodal disease, predicting prognosis after resection, and selecting candidates for adjuvant chemotherapy after resection. The current study also emphasizes the need to retrieve a larger number of lymph nodes than ever (for example, ≥6 nodes [12,18]) in resections for gallbladder cancer, not only for accurate staging but also for clearance of nodal disease.

## Conclusions

The number of positive lymph nodes better predicts the outcome after resection than either location of positive lymph nodes or LNR in gallbladder cancer, provided that lymph node evaluation is sufficient. Dividing the number of positive lymph nodes into three categories (0, 1 to 3, or ≥4) is valid for stratifying patients based on the prognosis after resection.

## Consent

Written informed consent was obtained from the patients for publication of this report.

## Abbreviations

AJCC, American Joint Committee on Cancer; DSS, Disease-specific survival; LNR, Lymph node ratio; TLNC, Total lymph node count.

## Competing interests

The authors declare that they have no competing interests.

## Authors’ contributions

YS conceived the study and drafted the manuscript. JS helped to draft the manuscript and performed statistical analyses. TW performed chart review and follow-up of the study cohort. TO helped with chart review and patient follow-up. YA provided histological data. KH was responsible for the whole study and participated in its coordination. All authors read and approved the final manuscript.
